# Phytochemistry and Application of White Mustard (*Sinapis alba*) in Medicine and Dentistry—A Narrative Review

**DOI:** 10.3390/molecules31040674

**Published:** 2026-02-15

**Authors:** Aniela Brodzikowska, Bartłomiej Górski, Konrad Michałowski

**Affiliations:** 1 Department of Conservative Dentistry, Medical University of Warsaw, ul. Binieckiego 6, 02-097 Warszawa, Poland; aniela.brodzikowska@wum.edu.pl; 2 Department of Periodontology and Oral Diseases, Medical University of Warsaw, 02-091 Warsaw, Poland

**Keywords:** *Sinapis alba*, white mustard, glucosinolates, allyl-isothiocyanate, sinigrin, antibacterial, antifungal, antioxidant, gingivitis, oral health, periodontitis, phytotherapy

## Abstract

White Mustard (*Sinapis alba*) seeds contain glucosinolates, mainly sinigrin and sinalbin. Isothiocyanate metabolites, together with flavonoids and tocopherols, present anti-inflammatory, antimicrobial, and antioxidant activities. This narrative review is a result of a literature search in PubMed, Scopus, and Google Scholar, spanning in vitro, in vivo. and clinical studies. The presented data highlight that mustard-derived products suppress pro-inflammatory cytokines such as TNF-α and inhibit a broad spectrum of pathogens at micromolar concentrations. In the largest (n = 113) double-blind dental trial to date, a white-mustard toothpaste reduced the mean value of Silness-Löe plaque index by −2.43 vs. −1.95 placebo and bleeding on probing by 30.6% vs. 26.8% within four weeks, while salivary *Streptococcus mutans* and *Porphyromonas gingival* colony counts decreased by 40%. A six-month follow-up study with a sinigrin-rich “Bamberka” extract confirmed these gains and selectively suppressed red-complex periopathogens. Clinical translation is limited by heterogeneous extraction methods, a lack of phytochemical standardization, and an unresolved allergenic risk linked to seed proteins Sin a 1 and Sin a 2. Mustard, therefore, emerges as a promising phytotherapeutic adjunct for controlling inflammation, infection, and oxidative stress, but widespread use awaits harmonized manufacturing guidelines, comprehensive allergological screening, and rigorously designed randomized trials benchmarked against chlorhexidine.

## 1. Introduction

Mustard plants (*Sinapis* spp.) are annual herbaceous species of the *Brassicaceae* (*Cruciferae*) family, cultivated throughout temperate regions of Europe and Asia. Crushed or macerated seeds have for centuries been used in traditional medicine for rubefacient poultices, warming liniments, and digestive aids. Contemporary studies confirm that mustard seed is a rich source of bioactive compounds with significant healing potential [1,2].

Interest in plant-derived substances as either complementary or alternative agents to conventional pharmacotherapy is steadily increasing. Mustard seeds, due to their wide availability, low cost, and systemic safety profile, are increasingly incorporated into functional food and health-related products. Especially when erucic acid remains below the 2% EFSA threshold, as in low-erucic cultivars such as “Bamberka” (<0.5% erucic acid) [3]. For phytotherapy, mustard can be recognized as a raw material with anti-inflammatory, antimicrobial, immunomodulatory, and potentially anticancer properties [4,5].

Hydrolysis of seed glucosinolates by endogenous myrosinase synthesizes isothiocyanates such as allyl isothiocyanates. The presence of flavonoids and tocopherols further enhances the antioxidant capacity. These compounds are known to participate in immune responses, free radical scavenging, and regulation of inflammatory and metabolic pathways [6,7,8]. Mustard seeds, therefore, serve as a source of functional oils, dietary supplements, and phytopharmaceuticals [6,7].

Recent studies have drawn attention to the possible role of mustard in dentistry, particularly in the prevention and adjunctive management of periodontal diseases and caries bacteria development. Seed extracts containing sinigrin and allyl-isothiocyanate have recently been formulated into experimental toothpastes, lowering the total counts of *Streptococcus mutans*, *Porphyromonas gingivalis,* and *Candida albicans* as well as reducing gingival bleeding by 20% in short pilot trials [8]. There is no mustard-based dental health product available. With the growing demand for plant-based oral-care products, the lack of mustard formulations leads to the need for this kind of research in dental medicine [6].

The molecular pathways by which glucosinolates and isothiocyanates disrupt mature periodontal biofilms or modulate host inflammatory signaling remain only partly disclosed. Equally uncertain is long-term safety: the allergenic 2S-albumins Sin a 1 and Sin a 2, together with the cumulative irritant potential of isothiocyanates, raise legitimate concerns about mucosal tolerance when these agents are used for periods longer than the 2–8 week horizons investigated so far. These aspects remain unclear. Therefore, detailed studies and extended safety trials are still needed before mustard-based oral formulations can be recommended for routine clinical use.

Several reviews have summarized mustard phytochemistry and its wide biological activity. *Sinapis semen*–focused pharmacology/toxicity overview by Dang et al., 2023 [9]. Wide family-level overview of *Brassicaceae* plants by Rahman et al. [10]. A precise description of the chemical activity of mustard constituents was presented by Shristy et al. [11]. A cross-spice antimicrobial review that also includes mustard was published by Mayekar et al. [12]. Tian et al. (2020 [13] focused on *Brassica juncea* and highlighted the predominance of sinigrin and its pharmacological activity. Nguyen et al. (2024) reviewed the mustard phenolic compound, drawing attention to the impact of technological processing on its content and function [14]. While these reviews provide an important context and valuable background, none of them examined the potential role of mustard in dentistry. This review addresses that gap.

Although this review is framed at the genus level (*Sinapis* spp.), only *S. alba* shows demonstrable agricultural and biomedical relevance; the remaining species (*S. arvensis*, *S. pubescens*, *S. allionii*, *S. integrifolia*) have marginal or no documented importance. Therefore, the synthesis focuses on *S. alba*.

This review aims to synthesize the current evidence on the phytochemistry, biological activities, and potential dental and medical applications of *Sinapis alba*. The article incorporates available data from in vitro and in vivo studies, preliminary clinical trials, and toxicology reports, identifying knowledge gaps that should guide further investigations.

## 2. Materials and Methods

This review synthesizes current evidence on the therapeutic potential of mustard (*Sinapis* spp.). The primary literature is heterogeneous (in vitro, in vivo, and early clinical trials), frequently uses non-standardized extracts, and includes only small randomized controlled trials (RCTs). The authors could not provide a systematic review because of the limited number of available randomized controlled trials, including only two studies, both conducted by the present research team. We consequently adopted a narrative review approach. A structured literature search was performed using electronic databases including PubMed, Scopus, and Google Scholar. Relevant articles published from 1980 to 30 June 2025 were identified. The following Boolean string was applied: (“mustard” OR “Sinapis”) AND (“glucosinolates” OR “isothiocyanates”) AND (“oral health” OR “dentistry” OR “periodontitis” OR “gingivitis”). Database filters were restricted to the publication period 1980–30 June 2025 and the English language. In Scopus, the results were additionally filtered down to articles, reviews, and conference papers with available abstracts. In Google Scholar, the results were sorted by relevance, and the first 200 records (20 pages) were screened. Screening was stopped when no additional potentially eligible records were found. Records excluded by database filters refer to automated exclusions applied before manual screening (publication date outside the prespecified range, non-English language, and records without abstracts). Duplicate removal was performed across databases. Reference lists of key reviews and included clinical papers were also checked to identify additional relevant studies.

Two reviewers independently screened titles and abstracts to assess full texts for eligibility. No protocol was registered, and no meta-analysis was conducted. The study quality was appraised qualitatively. For clinical studies, we considered randomization, allocation concealment, blinding, and comparator. For in vivo/in vitro, we consider extract characterization, replication, and statistical reporting.

## 3. Botanical and Chemical Characteristics of Mustard

### 3.1. Botanical Taxonomy

White mustard-*Sinapis alba* L. taxonomic classification:

- Kingdom: *Plantae*
- Phylum: *Magnoliophyta*
- Class: *Magnoliopsida*
- Order: *Brassicales*
- Family: *Brassicaceae*
- Genus: *Sinapis* L.
- Species: *Sinapis alba* L.
- Species: *Sinapis arvensis* L.
- Species: *Sinapis pubescens* L.
- Species: *Sinapis allionii*
- Species: *Sinapis integrifolia*

The presented taxonomic categorization is based on data obtained from the botanical database, The Plant List, and the GRIN Taxonomy Database provided by the United States Department of Agriculture (USDA) [15].

White mustard (*Sinapis alba*), an annual herb, typically reaching 30–60 cm in height, is characterized by pinnately lobed leaves and yellow flowers arranged in racemes. The plant is native to the Mediterranean region but is now widely cultivated in temperate climates around the world [16,17]. The seeds of *S. alba* are spherical and light-yellow to beige in color. They are a source of oil, the main pharmacologically active component of the plant. Their phytochemical profile differs from other cruciferous plants, especially due to the presence of sinalbin, a glucosinolate specific to this species [15].

Note that “black” and “brown” mustards (*Brassica nigra*, *B. juncea*) are colloquially termed “mustard”, but they do not belong to the *Sinapis* genus and are outside this review’s scope.

### 3.2. Chemical Composition

Mustard seeds, especially mustard seed oil, are a source of bioactive compounds rich with pharmacological and nutritional properties. The chemical profile of *Sinapis alba* seeds includes glucosinolates, isothiocyanates, fatty acids, proteins, flavonoids, essential oils, and minerals [18]. The range of diverse biological activities, including antimicrobial, anti-inflammatory, antioxidant, and lipid-lowering effects, is presented in Table 1.

**Figure 1:**
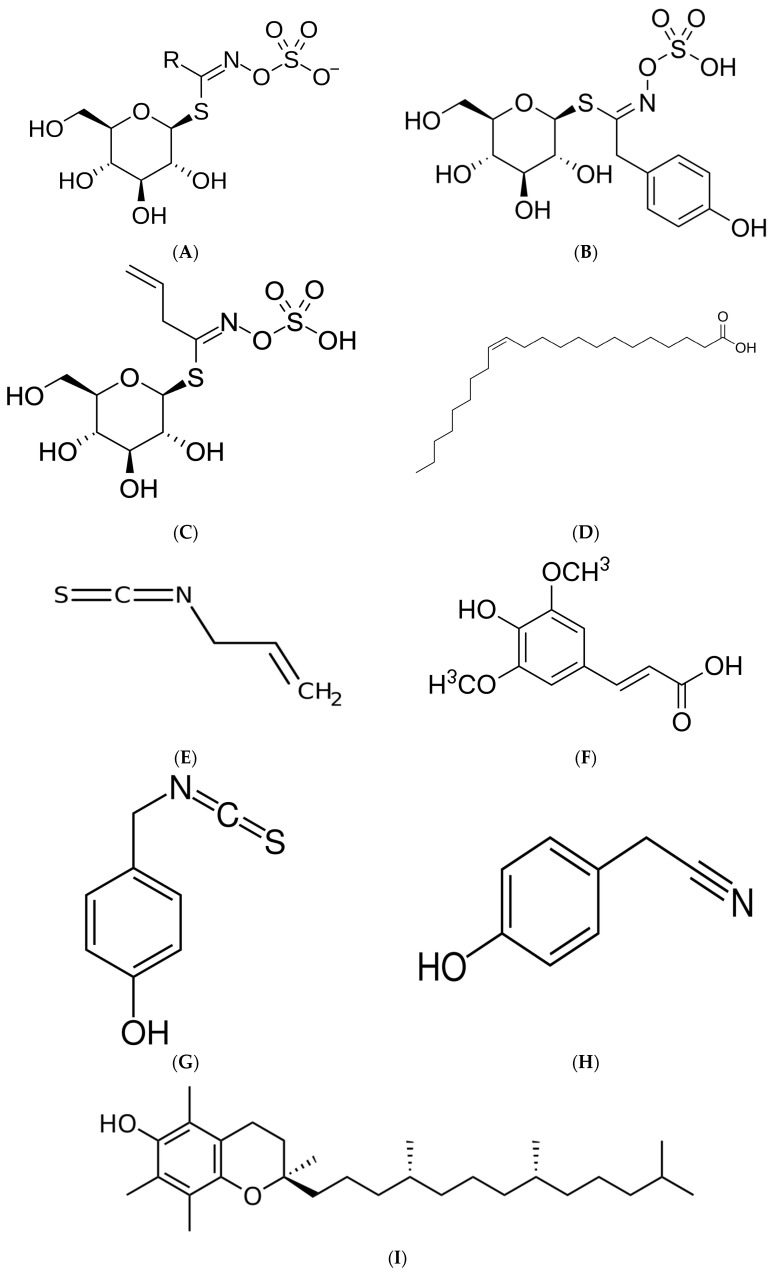
Key mustard-related molecules: (**A**) General glucosinolate structure (R-group varies depending on glucosinolate type), (**B**) Sinalbin (CAS: 20196-67-2), (**C**) Sinigrin (CAS: 3952-98-5), (**D**) erucic acid (cis-13-docosenoic acid) CAS: 112-86-7, (**E**) Allyl-isothiocyanate (AITC), (CAS: 57-06-7), (**F**) Sinapic Acid (CAS: 530-98-6) (**G**) p-Hydroxybenzyl isothiocyanate (p-HBITC), (CAS: 2086-86-4), (**H**) 4-Hydroxyphenylacetonitrile (CAS: 14191-95-8), (**I**) alpha-Tocopherol (CAS:59-02-9). CAS—Chemical Abstracts Service.

Among glucosinolates, sinalbin is the dominant compound in *S. alba*, while sinigrin occurs at higher levels in *Brassica nigra* and *Brassica juncea*. Upon enzymatic hydrolysis, glucosinolates are converted into isothiocyanates such as allyl-isothiocyanate, which exhibits antimicrobial activity against oral and gastrointestinal pathogens [4]. Mustard oil, extracted from the seeds, contains both beneficial unsaturated fatty acids, but may also contain erucic acid. In the European Union, maximum levels for erucic acid are set at 20 g/kg (2%) for most vegetable oils and fats, while higher levels are permitted for mustard oil (50 g/kg; 5%) due to naturally higher concentrations (Commission Regulation (EU) 2023/915) [7,21]. The chemical structures of key compounds are presented in Figure 1.

Mustard seeds contain 2S albumins, such as Sin a 1 and Sin a 2, which function as storage proteins and have been identified as major allergens [4]. Additionally, the presence of flavonoids like kaempferol and quercetin, as well as vitamin E (tocopherols), enhances the antioxidant profile of mustard, supporting its potential in managing oxidative stress-related diseases [2,20].

Certain isothiocyanates demonstrate antifungal activity. For example, AITC shows a minimum inhibitory concentration (MIC) of 0.125–0.5 mM against *Candida albicans* [22]. In vitro data suggest that AITC compromises membrane integrity, reduces epithelial adhesion, and prevents germ-tube formation, impairing tissue invasion [6,22]. These findings suggest the potential application of herbal antifungal rinses or gels in prosthodontic care for patients susceptible to fungal infection.

Comparative profiling of three *S. alba* cultivars, including the Polish variety Bamberka, demonstrated differences in glucosinolate and phenolic levels, further modulated by roasting [23].

Long-term safety remains insufficiently characterized. Mustard contains the 2S albumins Sin a 1 and Sin a 2, which are recognized food allergens capable of eliciting IgE-mediated and contact reactions. Future studies should assess the immunological risks associated with repeated mucosal exposure, particularly in children [24,25].

### 3.3. Species Overview Within Sinapis

Within the *Sinapis* Spp., white mustard (*S. alba*) is the only species with a substantiated agricultural and biomedical profile. The remaining taxa (*S. arvensis*, *S. pubescens*, *S. allionii, S. integrifolia*) appear to have marginal relevance. A preliminary PubMed scoping restricted to Title/Abstract pointed out that a vast majority of medically oriented records were related to the *S.* alba species.

*Sinapis alba* is the best-known species, *S. arvensis* has a mixed glucosinolate profile, and *S. nigra* is often considered to be a mustard plant. *Brassica juncea*, which is not a true *Sinapis*, is frequently included in reviews due to its strong pharmacological background and its role in traditional medicine.

The key difference lies in the dominant glucosinolates: *S. alba* produces mainly sinalbin, which hydrolyzes to p-HBITC. *S. nigra* and *B. juncea* are rich in sinigrin, the precursor of AITC. These differences are important because isothiocyanates vary in antimicrobial activity, irritant effect, and safety.

## 4. Pharmacological Activities and Potential Medical Applications

White mustard (*Sinapis alba)* is increasingly viewed as a promising adjunct in the prevention and management of diverse systemic disorders, a reputation that stems largely from its reservoir of glucosinolates and their enzymatically liberated derivatives-isothiocyanates. These molecules influence a wide range of physiological and pathophysiological processes [26].

### 4.1. Anti-Inflammatory Activity

Allyl-isothiocyanate inhibits the enzymes cyclooxygenase-2 (COX-2) and inducible nitric oxide synthase (iNOS). In animal models, they also lower the expression of pro-inflammatory cytokines, including TNF-α and IL-6 [27,28,29]. Central to this effect is activation of the Nrf2–Keap1, a master regulator of cellular antioxidant and anti-inflammatory defense [30].

At the molecular level, sinapic acid suppresses NLRP3 inflammasome activation. It decreases the levels of caspase-1 and IL-1β as well as attenuates NF-κB signaling. Mustard constituents also improve epithelial barrier readouts [9].

In LPS-stimulated macrophages, AITC reduced COX-2 and iNOS activity with IC_50_ values of ~8–15 μM, consistent with covalent modification of cysteine residues in Keap1 and activation of Nrf2 [30].

### 4.2. Anticancer Properties

Mustard-derived isothiocyanates have shown clear antineoplastic potential in both cell-culture and animal experiments. Reported actions include cell cycle arrest, promotion of apoptosis, and restriction of angiogenesis [31]. The supporting data were obtained from evaluations of colorectal, breast, and prostate cancer models [27,32,33]. A vast majority of evidence is preclinical.

The antitumor effects converge on apoptotic pathways, including the upregulation of Bax and downregulation of Bcl-2. They also reduce the protein expression of PTGS1, PTGS2, MMP-2, and MMP-9, and induce ferroptosis, reinforcing cell cycle arrest in hormone-responsive models [9,11].

In colorectal cancer HT-29 cells, AITC reduced viability with IC_50_ values around 15–25 μM. It is linked to apoptotic Bax/Bcl-2 modulation [33].

### 4.3. Antidiabetic Effects

Extracts from *Brassica juncea* and *Sinapis alba* lower fasting blood glucose and serum lipids, improve insulin sensitivity, and attenuate oxidative stress in diabetic animal models [34]. The proposed mechanisms include facilitation of glucose transporter activity, AMPK activation, and antioxidant activity of flavonoids [35].

In diabetic rat models, sinigrin-rich extract from *B. juncea* lowered fasting blood glucose by 20~30% and triglycerides by ~25%, likely via AMPK activation.

### 4.4. Antimicrobial Effects

Mustard oil and isothiocyanates present in the seeds act against a wide spectrum of microorganisms, including *Escherichia coli*, *Staphylococcus aureus,* and *Candida albicans*. Their activity stems from three complementary actions: disruption of microbial membranes, inhibition of respiration, and interference with quorum-sensing signaling [36,37,38]. Due to these multiple actions, mustard extracts are being explored both as natural food preservatives and adjuncts to antimicrobial therapy [39,40,41,42].

An in vitro study showed that incorporation of mustard seed extract into metal nanoparticles increased antimicrobial effects in vitro, indicating a potential strategy for improving the efficacy of natural agents [43].

Against oral pathogens, mustard-derived ITCs showed minimum inhibitory concentration (MIC) values of 0.0025–0.08 mg/mL and MBC 0.005–0.34 mg/mL. AITC inhibits growth with MIC 0.012–0.05 mg/mL [8,40].

To improve translational interpretability, we unified concentration reporting across studies where possible and explicitly relate in vitro MIC ranges (typically µM–mM) to concentrations used in oral formulations.

Recent experimental work has also shown that allyl-isothiocyanate at low concentrations (0.1%) almost completely inhibits *Streptococcus mutans* survival, growth, and biofilm formation, further confirming its anti-cariogenic potential [44].

### 4.5. Antioxidant Properties

Mustard seeds contain flavonoids such as kaempferol and quercetin, along with vitamin E (tocopherols). These compounds neutralize reactive oxygen species and shield cellular components from oxidative damage [45], thereby supporting cardiovascular and neuronal health and potentially slowing metabolic and neurodegenerative disorders [46].

Sinapic acid displayed DPPH scavenging with EC_50_ ~ 20–25 μM, while quercetin and kaempferol typically act in the 10–30 μM range, reflecting the role of hydroxyl and methoxy substitutions in radical scavenging [14].

In addition, p-hydroxybenzyl isothiocyanate improved the oxidative stability of soybean oil, indicating that isothiocyanates may also contribute to lipid protection beyond their antimicrobial role [47].

### 4.6. Neuroprotective Potential

Direct neuroprotective evidence for *S. alba* is currently scarce. Most data on neuroprotection within the *Brassicaceae* family come from other glucosinolate-rich plants (e.g., broccoli-derived sulforaphane). In preclinical models, such isothiocyanates activate Nrf2–Keap1 signaling, enhance antioxidant enzymes, and attenuate neuroinflammation [48].

These findings provide a mechanistic context and suggest that *S. alba*-derived isothiocyanates could potentially act through similar redox-sensitive pathways, but dedicated studies using standardized *S. alba* extracts or defined constituents are needed before any neuroprotective claims can be made [49].

Neuroprotective actions involve dampening oxidative stress and activating cAMP/PKA/CREB and BDNF/TrkB/ERK signaling [9,11].

Recent trials using broccoli rich in glucoraphanin showed significantly lower levels of oxidative-stress biomarkers and improvement in lipid profiles. Therefore, thioglucosides may be considered to be an alternative preventive or adjuvant therapy for neurodegenerative disease [50]. Key biological properties are combined and presented in Table 2.

### 4.7. Mustard Application in Dentistry

Clinical evidence for mustard-derived products in dentistry is emerging but remains limited. Most data concern plaque control and gingival inflammation, with a small number of clinical trials evaluating mustard extract toothpastes and several smaller studies testing mustard oil or extract-based formulations in specific indications.

In a double-blind randomized controlled trial in patients with gingivitis (n = 113), brushing twice daily for 4 weeks with a toothpaste containing S. alba extract improved clinical indices, including Silness-Löe plaque index (PI), approximal plaque index (API), according to Lange et al., gingival index (GI), and bleeding on probing (BoP). Compared with placebo toothpaste, the experimental formulation reduced PI by 2.43 and BoP by 30.6%. Microbial outcomes assessed using the Caries Risk Test (CRT, Ivoclar Vivadent), reporting *Streptococcus mutans* and *Lactobacillus* spp. salivary loads were significantly lowered.

Besides toothpaste formulations, other delivery forms (e.g., gels, local drug-delivery systems, and oil pulling) have been explored in dentistry, but the evidence remains heterogeneous. An in vitro study reported reduced growth of oral bacteria and *Candida albicans* after application of mustard gel. In a separate single-blind clinical study, a sinigrin-containing gel applied twice daily for 14 days reduced plaque index by 30% and gingival bleeding by 22%. These outcomes are not directly comparable with the 4-week toothpaste RCT because of differences in formulation, dosing regimen, and study design.

In small-scale randomized controlled trials, toothpaste enriched with mustard extract shows a reduction in dental plaque accumulation and periodontal inflammation parameters [51]. An in vitro study by Devika et al. demonstrated a significant reduction in the growth of oral bacteria and *Candida albicans* after application of mustard gel [30]. Preliminary clinical evidence supports the use of mustard-derived compounds in oral care. In a single-blinded study, sinigrin-containing gel applied twice daily for 14 days reduced the plaque index by 30% and gingival bleeding by 22%.

A pilot clinical trial by Bajpai, using a mustard seed extract hydrogel as a local drug-delivery system in periodontitis, demonstrated adjunctive benefits to nonsurgical periodontal therapy, with reductions in plaque and bleeding scores [52].

Beyond toothpaste formulations, a double-blind randomized trial of Kavala Gandusha (oil pulling) compared mustard oil with rice bran oil in gingivitis patients and found improvements in plaque and gingival indices as well as salivary pH, although rice bran oil showed slightly greater efficacy [53].

In endodontics, mustard gels have also been tested as an adjuvant in root canal medicaments. In vitro clinical study found activity against *Enterococcus faecalis* comparable to chlorhexidine, although larger trials are still needed [54].

A clinical study of chewing gum containing AITC from mustard seed extract reported short-term reductions in volatile sulfur compounds associated with oral malodor [55].

The key characteristics of dental studies are summarized in Table 3 to facilitate comparison and highlight current gaps in standardization. Overall, current dental studies are early-stage and heterogeneous. Future work should prioritize standardized phytochemical profiling, head-to-head comparisons with gold-standard antimicrobials (e.g., chlorhexidine), and systematic monitoring of mucosal tolerability and allergenicity during prolonged exposure.

These findings suggest that mustard-based formulations may have clinical utility as adjuncts in periodontal therapy. The clinical outcome of this finding was observed in an RCT evaluating a reduction in the development of *Lactobacillus spp.* and *Streptococcus Mutans* in saliva [56]. A comparison of AITC with chlorhexidine, a properly examined and well-documented antibacterial substance, was presented in Table 4.

## 5. Erucic Acid, “Bamberka” Cultivars, Allergy, and Safety Measures

### 5.1. Erucic Acid

Erucic acid is a monounsaturated omega-9 fatty acid (cis-13-docosenoic acid) presented in Figure 1 that naturally occurs in the seed oil of mustard (*Sinapis* spp.) and rape seed (*Brassica napus*). While physiologically tolerated at low levels, high dietary intakes have been shown to produce adverse metabolic and cardiotoxic effects in animal studies, most notably myocardial lipidosis with excessive lipid deposition and mitochondrial alterations in cardiomyocytes [6,57].

Considering these data, regulatory bodies such as the European Food Safety Authority (EFSA) and the Codex Alimentarius Commission have set strict limits on the allowable content of erucic acid in edible oils. EFSA guidelines for food-grade products allow a maximum of 2% of total fatty acids [6,58].

Safety concerns have driven plant-breeding and biotechnology programs aimed at producing low- or zero-erucic cultivars. Such lines are critical not only for human and animal nutrition but also for the development of functional therapeutic preparations [3,59].

### 5.2. The “Bamberka” Cultivar

In 2006 The Plant Breeding and Acclimatization Institute-National Research Institute (PBAI-NRI) (Poznań, Poland) developed and presented a low-erucic white-mustard cultivar “Bamberka”. This development addresses the demand for safer raw materials [3]. The oil pressed from seeds contains less than 0.5% erucic acid and more than 63% oleic acid, a profile regarded as cardioprotective [60].

Beyond its improved lipid composition, “Bamberka” also offers agronomic advantages: it demonstrates positive activity against soil pathogens, helps maintain soil structure, and constitutes a useful rotation crop. From the medical perspective, its low-erucic content and favorable safety record make the cultivar an attractive source for topical oral-care ingredients [11]. The introduction of “Bamberka” brings the field closer to standardized, reproducible starting raw materials for the preparation of mustard-based products.

### 5.3. Allergy

Because mustard is a recognized allergen, great caution is required when formulating consumer products, particularly for patients with atopy or food allergies. Individuals with confirmed mustard allergy, or with known cross-reactivity to other *Brassicaceae* (e.g., Cabbage, broccoli, cauliflower) or to weed pollens such as mugwort and ragweed, should be excluded from treatment. In patients with asthma, eczema, or multiple food allergies, a careful allergy history is essential [24,61]. If any doubt remains, patch testing or referral to an allergy specialist is required before use [62,63,64].

Mechanistic studies have clarified that the mustard allergen Sin a 1 activates epithelial and dendritic cells to drive type-2 immune responses, reinforcing its role in IgE-mediated allergy [65].

### 5.4. Safety Measures

All mustard-containing dental products should be clearly labelled in line with international allergen regulations (e.g., EU 1169/2011) [40]. For ingestible products, erucic acid content should be controlled (preferably using low-erucic cultivars) and compliance with maximum levels should be verified. For topical/mucosal dental products, systemic exposure is expected to be lower, but accidental ingestion and repeated mucosal contact still warrant careful safety monitoring. Clinical studies should explicitly report tolerability endpoints (oral burning, mucosal erythema/ulceration, contact dermatitis), implement exclusion criteria for known mustard allergy, and provide a clear adverse-event reporting framework. Preparations intended for mucosal application ought to employ standardized, purified extracts to maximize safety [46]. Future work should focus on producing hypoallergenic derivatives—for example, by enzymatic digestion or selective extraction to lower Sin a 1 and Sin a 2 levels [47].

Contact with mustard may cause dose- and time-dependent skin irritation and blistering, primarily due to the presence of allyl-isothiocyanate. Reports on whether stronger skin reactions correlate with better outcomes of acupoint patches are inconsistent; factors such as application time, seed processing (raw or processed), and plant species contribute to this variability. Standardized protocols and explicit reporting of skin outcomes are recommended to ensure translational relevance.

Formulations should therefore control free isothiocyanate release (e.g., using encapsulation or protein-reduced extracts) and define realistic contact time and dosing. Because the degree of protein removal and the release kinetics of isothiocyanates may also influence antimicrobial efficacy, future studies should report both allergen content (e.g., Sin a 1/Sin a 2) and active markers (sinalbin/sinigrin and isothiocyanates) to enable benefit-risk assessment and replication.

Mustard-based agents could become valuable adjuncts in dentistry, but only if their immunological safety is established through well-designed clinical trials and ongoing pharmacovigilance, especially in children and immunocompromised patients.

## 6. Discussion

The growing number of experimental and clinical studies indicates that mustard (*Sinapis* spp.) possesses biomedical potential. Its complex chemical profile, consisting of glucosinolates, isothiocyanates, and phenolic compounds, provides a wide range of biological activities [1,2]. In both oral and general health contexts, mustard-based preparations are recognized for their capacity to modulate inflammation, inhibit pathogenic microorganisms, and support mucosal resilience [66].

Unfortunately, current data are fragmentary and limited. Most preclinical studies involved isolated phytochemicals or unstandardized extracts, which present difficulties for interpretation and replication. In fact, because many knowledge gaps remain unclear, the clinical application of mustard-based products remains mostly theoretical.

In the domain of dental medicine, early-phase investigations have indicated favorable outcomes regarding plaque control and gingival health [51]. Moreover, only a few human trials have been conducted, with limited sample sizes and without comparisons to gold-standard treatments [67]. Wider application of mustard in dental protocols has to be validated through comparative clinical trials. A comparison of mustard thioglucosides with well-established gold standards like chlorhexidine must be conducted. The study should assess patient-reported outcomes, formulation acceptability, and long-term mucosal tolerance.

Allergy potential is important and must be taken into consideration. Although rare in the general population, hypersensitivity reactions to mustard are well-documented and may be clinically significant in vulnerable groups [61,68,69,70]. The development of hypoallergenic extracts, alongside strict labelling and preclinical safety assessments, should be considered to be an important step in the clinical implementation of mustard-based products.

Several key areas have been identified as paths for future investigation. Current studies should involve standardization and optimization of phytotherapeutic formulations. The development of standardized extracts with defined phytochemical profiles, particularly those derived from low-erucic acid cultivars like “Bamberka”, will be essential for future clinical applications and product development.

Important knowledge gaps reduce the clinical translation of mustard-based products. Firstly, the molecular pathways by which allyl-isothiocyanate disrupts multispecies oral biofilms have been described largely by inference; in situ studies that delineate these signaling cascades are required to optimize therapeutic targeting and minimize off-target effects. Secondly, long-term safety remains insufficiently characterized: no longitudinal trial has monitored mucosal integrity or systemic distribution beyond 12 weeks of continuous exposure, leaving the risks associated with chronic use undefined. Finally, in the future, formulation science has yet to keep pace with biological possibilities, like dose-delivery platforms such as muco-adhesive gels, nano-emulsions, and slow-release varnishes, which have not been compared directly for efficacy, stability, or patient acceptability.

Particular attention should be paid to vulnerable populations, including children, pregnant women, and patients with pre-existing allergies or autoimmune disorders.

## 7. Conclusions

White mustard (*Sinapis alba*) provides a rich blend of glucosinolates, isothiocyanates, flavonoids, and tocopherols. This composition explains the antimicrobial, anti-inflammatory, and antioxidant activities observed in preclinical models.

### 7.1. Clinical Implications

Preliminary clinical studies have reported benefits in plaque control, gingivitis reduction, and as an adjunct in the treatment of oral infections. Antifungal properties might be used for providing prophylactic protocols. Implementing mustard as a raw material to produce plant-based oral-care products (mouth rinses and toothpastes) should be considered after performing large-scale randomized controlled trials and fully characterizing its safety.

### 7.2. Research Limitations

The optimal therapeutic dose is unknown, and it is still unclear what proportion of mustard constituents is responsible for the observed effect. Clinical evidence comes from only two small, short-term clinical trials on humans. Available data come from in vitro models, which constitute insufficient scientific evidence. Long-term outcomes and the risk of contact allergy for human beings remain unclear.

### 7.3. Recommendation for Future Research

A wide range of allergology and toxicology studies should be performed to establish safety before mustard-based products can be adopted in routine care. Conducting further RCTs is important to confirm clinical efficacy and safety.

Furthermore, the present review does not cover minor *Sinapis* species due to their limited biomedical literature. Future studies may revisit this decision if new primary data emerge.

### 7.4. Outlook

Bridging traditional phytotherapy with evidence-based medicine will establish mustard as a raw material in modern medicine. Conducting high-quality clinical trials and developing hypoallergenic, standardized formulations is important to translate mustard into safe and effective therapeutic options.

## Figures and Tables

**Table 1:** Selected bioactive components of mustard seeds and their biological activities.

Bioactive Component	Main Fractions	Biological Activity	References
Glucosinolates	Sinalbin sinigrin	Precursors of isothiocyanates	Fahey et al. [1]
		Antimicrobial	
		anticancer effects	
Isothiocyanates	Allyl-isothiocyanate	Cytotoxic	Fahey et al. [1] Lin et al. [5]
	p-hydroxybenzyl isothiocyanate	anti-inflammatory Pungent flavor Antiseptic properties	
Fatty acids	Erucic acid	Hypolipidemic erucic acid is cardiotoxic at high doses	EFSA [6,7]
	Oleic acid		
	Linoleic acid		
	Palmitic acid		
Proteins	2S-albumins (Sin a 1, Sin a 2) 7S and 11S globulins	Potential allergens emulsifying properties	Lin et al. [4,15,19]
Flavonoids and Phenolics	Kaempferol Quercetin Rutin	Antioxidant Radical scavenging	Peterson et al. [20]
Minerals	Potassium Calcium Iron Magnesium	Metabolic and antioxidant roles	USDA [15,19]

**Table 2:** Biological properties and potential medical applications of mustard compounds.

Biological Activity	Main Compounds	Mechanism of Action	Potential Application	Level of Evidence
Anti-inflammatory	Isothiocyanates AITC p-HBITC	Inhibition of: COX-2, iNOS, TNF-α, IL-6, Nrf2 activation	Anti-inflammatory application	In vivo In vitro
Anticancer	Isothiocyanates	Induction of the cell apoptosis cycle	Cancer	In vivo In vitro
	Flavonoids			
Hypoglycemic Hypolipidemic	Polyphenols Fatty Acids Isothiocyanates	Activation of antioxidant effects Insulin sensitization	Type 2 diabetes Metabolic syndrome	In vivo
Antibacterial	AITC Essential Oils	Disruption of the membrane, enzyme inhibition, anti-quorum-sensing	Oral infections, Skin infections	In vitro
Antioxidant	Kaempferol Quercetin Vitamin E	scavenging, lipid peroxidation inhibition	Cardiovascular protection, neuroprotection	In vitro
Immunomodulatory	Glucosinolates Tocopherols	Modulation of cytokine production, T cell activity	Autoimmune diseases, mucosal healing	In vitro In vivo
Antifungal	AITC	Disruption of fungal membranes, hyphal inhibition	Oral candidiasis, dermatomycoses	In vitro In vivo

Abbreviations are listed and explained in the abbreviations section.

**Table 3:** Summary of dental studies using mustard-derived products.

Study	Study Design	Species and Plant Part	Form and Dosage	Comparator	Timeframes	Key Outcomes
Michałowski et al., 2025 (Dent. J.) [56]	Double-blinded RCT, patients with gingivitis, n = 113	*Sinapis alba*, cv. “Bamberka”; Fragmented seeds and extract	Toothpaste with 1% *Sinapis alba* extract	Placebo toothpaste	4 weeks,	PI −2.43 vs. −1.95; BoP −30.6% vs. −26.8%; reduced salivary *S. mutans* & *Lactobacillus* spp. loads vs. placebo.
Michałowski et al., 2024 (Int. J. Mol. Sci.) [51]	Single-blinded RCT, patients with gingivitis, n = 66, stratified by DMFT/CPI	*Sinapis alba*, cv. “Bamberka”; Fragmented seeds and extract	Toothpaste with 1% *Sinapis. alba* extract	Placebo toothpaste	12 months, follow-up at 6 and 12 months	Significant reductions in PI, API, and BoP. The largest changes occurred within the first 6 months.
Bajpai et al., 2024 (J. Clin. Diagn. Res.) [52]	Pilot clinical study, patients with periodontitis	*Brassica nigra*; Seeds	Mustard seed extract hydrogel as a local drug-delivery adjunct	Conventional nonsurgical periodontal therapy	14 days,	Adjunctive reductions in plaque and gingival indices reported; limited standardization details.
Ahuja et al., 2023 (RGUHS J. Dent. Sci.) [53]	Double-blinded trial; patients with gingivitis; n = 45	*Sinapis alba*, Seed Oil	Kavala Gandusha (oil pulling) with mustard oil	Rice bran oil	7 and 14 days	Both improved plaque formation and gingival indices. Mustard oil reduces the number of bacteria colonies.
Bajpai et al., 2023 (JCPSP) [30]	In vitro endodontic study	*Sinapis alba*, Seed extract	Mustard gel	2% chlorhexidine (gel)	Not applicable	Reported activity against *E. faecalis* was comparable to 2% CHX in that model.
Tian et al., 2013 (Am. J. Dent.) [55]	Short-term clinical testing; n = 10	*Sinapis alba*, Seeds	Chewing gum with AITC	Placebo gum	Single-use, minutes	Short-term reduction in volatile sulfur compounds (oral malodor surrogate).

**Table 4:** Comparison of allyl-isothiocyanate (AITC) and chlorhexidine (CHX).

Properties	Allyl Isothiocyanates (AITC)	Chlorhexidine (CHX)
Antibiofilm activity and substantivity	Antibiofilm effects reported in intro studies. Substantivity in the oral cavity is not well characterized.	Well-established plaque inhibition and high substantivity in oral surfaces.
Advantages	Potential dual antimicrobial and anti-inflammatory effects may be incorporated into natural oral-care formulations.	Predictable dosing and robust efficacy for short-term plaque and antiseptic control
Antimicrobial spectrum	Broad Gram-positive/Gram-negative bacteria, yeasts, *Candida albicans.*	Broad Gram-positive/Gram-negative bacteria limited against spores, fungi, and viruses
Origin	natural–plant hydrolysis product.	Synthetic antiseptic
Cost	Low raw material cost, potentially low cost depends on standardization, manufacturing, and quality control.	Low to moderate, widely manufactured
Adverse effect and limitations	Irritant potential, highly allergenic seed proteins.	Tooth and tongue staining, calculus build-up taste alteration, not recommended for long-term and continuous use
Availability	Limited and experimental use, lack of commercial products with defined standardization.	Widely available commercial, OTC, and professional products
Overall Evidence level for antibacterial and antibiofilm efficacy	Low, limited to in vitro and in vivo studies and early clinical studies.	High, well-established RCTs, Systematic reviews, and clinical evidence

## Data Availability

The raw data supporting the conclusions of this article will be made available by the authors on request.

## Abbreviations

The following abbreviations are used in this manuscript:

AITC	Allyl-isothiocyanate
AMPK	AMP-activated protein kinase
API	Approximal plaque index
BDNF	Brain-derived neurotropic factor
BoP	Bleeding on Probing
CAS	Chemical Abstract Service
CHX	Chlorhexidine
COX-2	Cyclooxygenase-2
CPI	Community Periodontal Index
CREB	cAMP response element-binding protein
DMFT	Decayed, Missing, and Filled Teeth (index)
DPPH	2,2-Diphenyl-1-picrylhydrazyl
EC50	Half Maximal Effective Concentration
EFSA	European Food Safety Authority
GI	Gingival Index
IC50	Half maximal inhibitory concentration
IgE	Immunoglobulin E
IL-1	Interleukin 1
IL-6	Interleukin 6
iNOS	Inducible nitric oxide synthase
Keap1	Kelch-like ECH-associated protein 1
MBC	Minimum Bactericidal Concentration
MIC	Minimum Inhibitory Concentration
Nrf2	Nuclear factor erythroid 2-related factor 2
PI	Plaque Index by Silness-Löe
PKA	Protein kinase A
p-HBITC	p-Hydroxybenzyl isothiocyanate
RCT	Randomized Control Trials
ROS	Reactive oxygen species
TNF-α	Tumor necrosis factor alpha
TrkB	Tropomyosin Receptor Kinase B
USDA	United States Department of Agriculture
